# Selective Targeting of αvβ5 Integrin in HepG2 Cell Line by RGDechi15D Peptide

**DOI:** 10.3390/molecules25184298

**Published:** 2020-09-19

**Authors:** Domenica Capasso, Annarita Del Gatto, Daniela Comegna, Luigi Russo, Roberto Fattorusso, Michele Saviano, Sonia Di Gaetano, Laura Zaccaro

**Affiliations:** 1 CESTEV, University of Naples “Federico II”, 80145 Naples, Italy; domenica.capasso@unina.it; 2 CIRPeB, University of Naples “Federico II”, 80134 Naples, Italy; annarita.delgatto@unina.it (A.D.G.); Roberto.FATTORUSSO@unicampania.it (R.F.); msaviano@unina.it (M.S.); 3 Institute of Biostructures and Bioimaging, CNR, 80134 Naples, Italy; daniela.comegna@unina.it; 4 Department of Environmental, Biological and Pharmaceutical Sciences and Technologies, University of Campania “Luigi Vanvitelli”, 81100 Caserta, Italy; LUIGI.RUSSO2@unicampania.it; 5 Institute of Crystallography, CNR, 70126 Bari, Italy

**Keywords:** αvβ5 Integrin, peptide antagonist, tumor cell adhesion, tumor cell invasion, HCC, angiogenesis

## Abstract

Recently, the research community has become increasingly concerned with the receptor αvβ5, a member of the well-known integrin family. Different ongoing studies have evidenced that αvβ5 integrin regulates not only physiological processes but also a wide array of pathological events, suggesting the receptor as a valuable biomarker to specifically target for therapeutic/diagnostic purposes. Remarkably, in some tumors the involvement of the receptor in cell proliferation, tumor dissemination and angiogenesis is well-documented. In this scenario, the availability of a selective αvβ5 antagonist without ‘off-target’ protein effects may improve survival rate in patients with highly aggressive tumors, such as hepatocellular carcinoma. We recently reported a cyclic peptide, RGDechi15D, obtained by structure-activity studies. To our knowledge it represents the first peptide-based molecule reported in the literature able to specifically bind αvβ5 integrin and not cross react with αvβ3. Here we demonstrated the ability of the peptide to diminish both adhesion and invasion of HepG2 cells, an in vitro model system for hepatocellular carcinoma, to reduce the cell proliferation through an apoptotic process, and to interfere with the PI3K pathway. The peptide, also decreases the formation of new vessels in endothelial cells. Taken together these results indicate that the peptide can be considered a promising molecule with properties suited to be assessed in the future for its validation as a selective therapeutic/diagnostic weapon in hepatocarcinoma.

## 1. Introduction

Integrins are transmembrane receptors able to dictate cellular responses to a variety of inputs thanks to their capacity to differentially recognize distinct environments. To allow for this flexibility, integrins are comprised of 18 α and 8 β subunits that pair to form at least 24 different functional heterodimeric receptors. They can transduce extracellular stimuli resulting in an extensive range of downstream effects on cell adhesion, migration, proliferation, differentiation and apoptosis [1]. In vivo studies have shown that in various types of cancers the expression of some integrins on the surface of neoplastic cells is frequently up-regulated. The αv subunit, which forms heterodimers with β1, β3, β5, β6 or β8 subunits, is able to recognize more extracellular matrix (ECM) ligands and growth factors thanks to the RGD sequence.

The availability of ligands able to discriminate among different integrin subtypes, sharing sequence and structure homology but different biological effects, is a desirable aim in the biomedical field. Over several decades the scientific community has focused on different integrins such as αvβ3, α5β1 and αvβ6 and little attention has been paid to integrin αvβ5 whose relevance has been assessed from several ongoing studies. αvβ5 integrin binds mainly the ECM protein vitronectin [2,3] and has the ability to control different biological and pathological events, thus representing one of the more intriguing integrins. It has been known for years that integrins are commonly used as receptors by many human viruses [4], and αvβ5 actually seems to play a critical role in multiple aspects of infection pathogenesis [5,6]. Viral proteins with the RGD motif promote infection by binding integrin heterodimers [4,7], thus activating PI3K/AKT or MAPK signaling pathways which promote virus entry and infection of the host cell [8]. Moreover, in addition to the well-established contribution of αvβ5 integrin to angiogenesis [9,10,11], much evidence supports the crucial role of this integrin in promoting cancer cell migration and invasion. It seems that αvβ5 mediates the early steps of liver metastasis formation of colon carcinomas through different mechanisms [12,13]. Additionally, there is substantial evidence for interplay between αv integrins and tissue growth factor (TGF-beta) during the pathological epithelial-to-mesenchymal transition that occurs in many types of cancer [14,15,16,17,18,19], which is the reason why anti-integrin therapeutics are indeed under development as treatment for TGF-beta-related disorders [20].

Hepatocellular carcinoma (HCC) is a primary malignancy of the liver that usually develops from a background of liver fibrosis and inflammation. It is associated with a high propensity for vascular invasion and metastasis, which accounts for its poor prognosis [21,22]. The high mortality rate is due to the advanced stage and frequent recurrence after surgical resection, and most patients are limited to surgical treatment or chemotherapy [23]. Consequently, to improve the survival rate, novel and effective therapeutic strategies are needed. Recently, Hoshino et al. proved that exosomal β5 integrin regulates liver tropism associated with liver metastasis in several tumors [12,13,24]. In a more recent study, Lin et al. demonstrated that β5 integrin is highly expressed in HCC tissues, facilitating cancer cell growth and promoting cell migration [25]. In human HCC patients, fibrinogen [26] activates hepatic stellate cells (HSC) [27] and liver specific mesenchymal cells by binding αvβ5 integrin [28] widely expressed by neoplastic cells to promote liver disease progression and tumor metastasis [29]. Recently Yan et al. stated that treatment with cilengitide peptide, an αvβ3/αvβ5 integrin antagonist, significantly blocked HSC activation and function and reduced proliferation of oncogenic hepatocytes and progression of liver fibrosis [30]. Thus, it is definitively clear that β5 integrin can be considered a diagnostic biomarker and a potential therapeutic target in HCC. 

To date only αvβ3/αvβ5 and αvβ3/α5β1 antagonists have been reported in the literature, whilst selective αvβ5 peptide antagonists are still missing. Selective modulation of αvβ5 integrin is highly desirable to allow targeted specific therapy and potentially to limit side-effects. This aspect, despite the availability of structural model of the head group of αvβ5 integrin constructed by homology modeling, is further complicated by the lack of the high-resolution 3D structure [31].

In the last few years, our research activities have mainly focused on the identification of integrin selective peptide ligands by rational design [32,33,34,35]. In 2016 we reported NMR and computational studies on the αvβ3 selective peptide, RGDechi, aiming to identify the molecular basis that regulates receptor recognition mechanism. By this combined approach we also demonstrated that the substitution of the key homocitrulline residue with aspartic acid shifts the receptor binding selectivity from αvβ3 to αvβ5 integrin, thus identifying a novel and selective αvβ5 ligand, named RGDechi15D [36].

Here we reported the in vitro characterization of this peptide on the HepG2 cell line, one of the well-known model systems for HCC [37], evaluating its ability to selectively bind αvβ5 integrin and to interfere with angiogenesis, cell migration, invasion and proliferation.

## 2. Results and Discussion

### 2.1. Integrin Expression on HepG2

**Figure 1:**
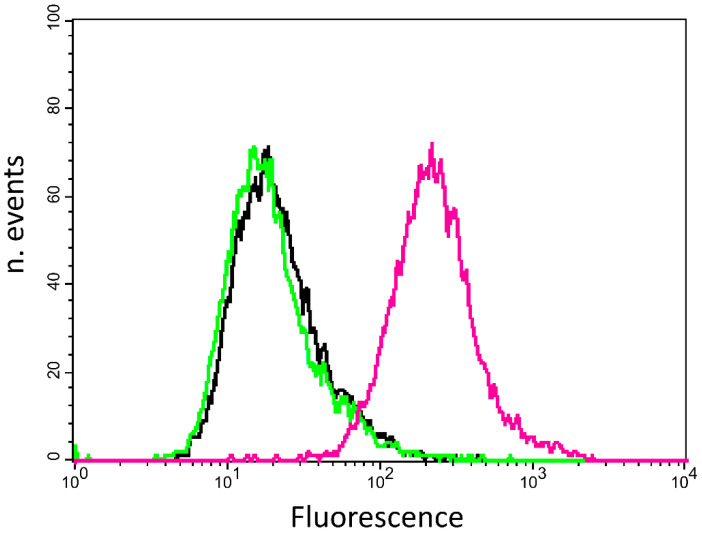
Quantification of αvβ3 and αvβ5 receptors on HepG2 cell surface. Cells were stained with phycoerythrin (PE)-anti-αvβ5 antibody (magenta curve) or PE-conjugated anti-αvβ3 antibody (green curve) or PE-control isotype (black curve). The histogram is representative of three independent experiments.

The expression levels of αvβ5 and αvβ3 integrins on surface of HepG2 were determined by means of quantitative cytofluorimetric assay using phycoerythrin (PE) conjugated antibodies. To maintain surface marker integrity, cells were detached using 0.1 mM EDTA in PBS. Data indicate that HepG2 cells show an average surface expression of 1.7 × 10^4^ ± 1000 αvβ5 (Figure 1), while they do not present a significant amount of αvβ3 receptors. The good level of αvβ5 expression together with the absence of αvβ3 make this cell line a suitable model to study the biological behavior of a selective αvβ5 molecule. 

### 2.2. Effect of RGDechi15D on Cell Adhesion

Cell adhesion assays were performed in order to confirm the capability of the peptide to bind only αvβ5 and not to cross react with αvβ3 or α5β1, unlike the current peptide-based molecules reported the in literature which are not able to discriminate between these integrins.

**Figure 2:**
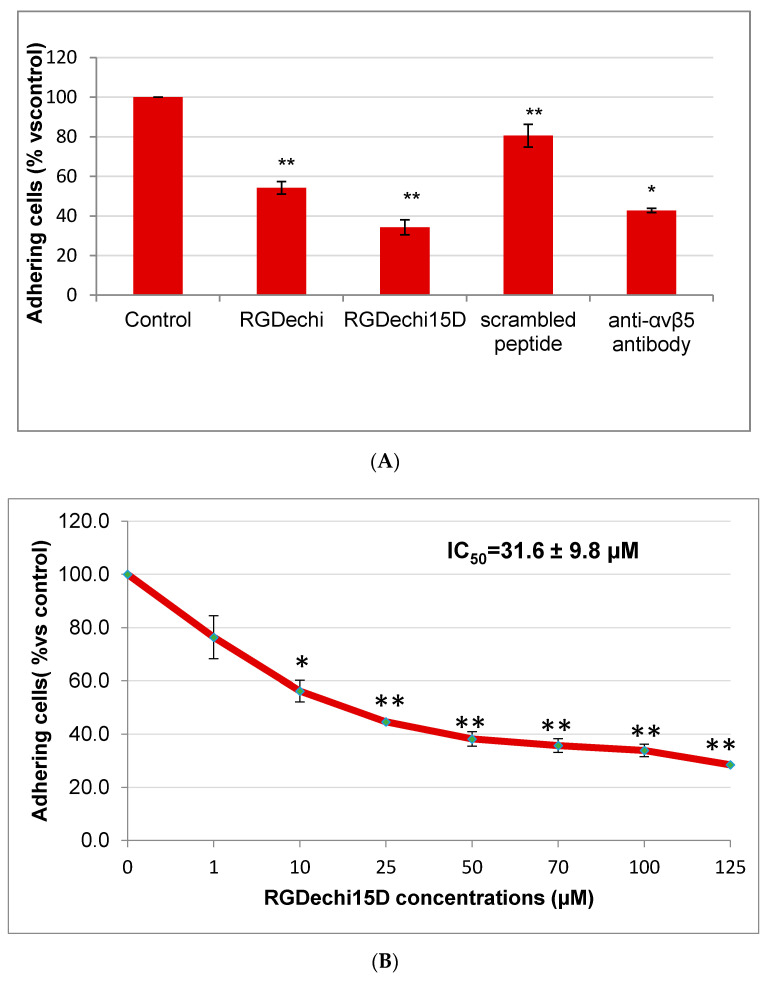
Inhibition of cell adhesion on vitronectin coated plates. (**A**) HepG2 cells were pre-incubated with peptides (50 µM) or anti-αvβ5 antibody (10 µg/mL) or (**B**) with increasing concentrations of RGDechi15D, for 30 min at 4 °C and then seeded on vitronectin coated plates at 37 °C. Cell adhesion was evaluated after 1 h of incubation using crystal violet reagent. Results are presented as the percentage of adherent cells with respect to the control (untreated cells) and are expressed as means ± standard error (SE) of at least three independent experiments performed in triplicate. Statistical significance was analyzed using Student’s t test, unpaired, two-sided (** *p* < 0.01, * *p* < 0.05).

**Figure 3:**
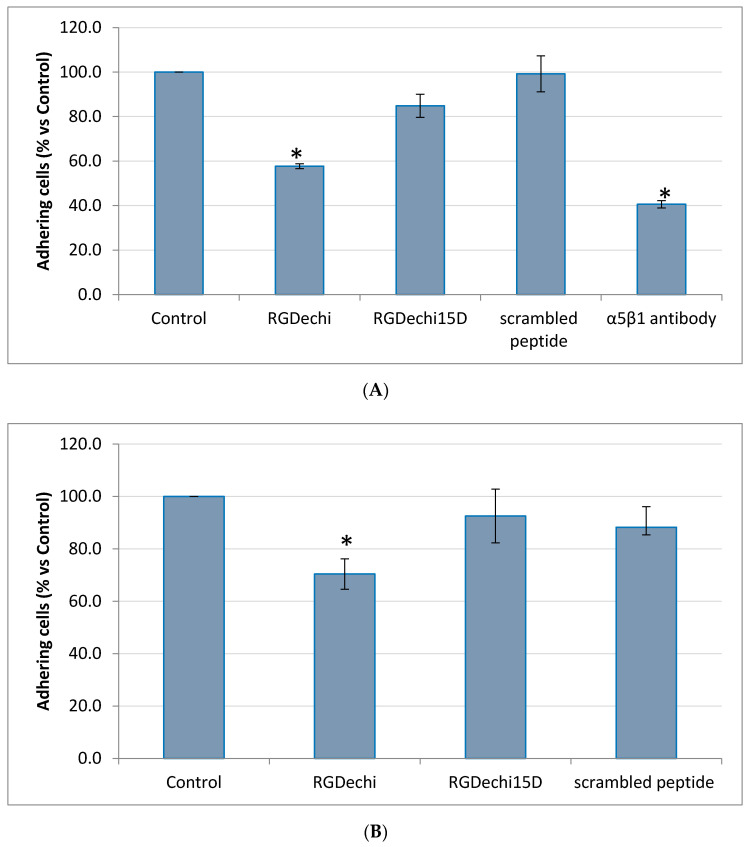
(**A**) Effect on K562 cell adhesion on fibronectin coated plates. Cells were pre-incubated with 50 µM RGDechi15D or RGDechi (used as positive control) or 10 μg/mL anti-αvβ5 antibody, for 30 min at 4 °C and then seeded on fibronectin coated plates at 37 °C. Cell adhesion was evaluated after 1 h of incubation using crystal violet reagent. (**B**) Effect on K562 adhesion on α5β1 antibody coated plates. K562 were pre-incubated with peptides (50 µM) for 30 min at 4 °C then seeded on anti-α5β1 coated plates. The results are presented as the percentage of adherent cells respect to the control (untreated cells) and are expressed as means ± SE of at least three independent experiments performed in triplicate. Statistical significance was analyzed using Student’s t test, unpaired, two-sided (* *p* < 0.05).

RGDechi15D, RGDechi and scrambled peptides were synthesized as previously reported [35,36]. The effect of RGDechi15D on HepG2 adhesion to vitronectin, a matrix able to recognize both αvβ3 and αvβ5 integrins [38,39,40], was analyzed by crystal violet assay. To prevent receptor internalization, the cells were pre-incubated at 4 °C with peptides or an anti αvβ5 integrin antibody; the treatment occurred in an appropriate adhesion buffer containing divalent cations essentials for receptor binding [36]. Then, cells were seeded onto vitronectin. As shown in Figure 2A, RGDechi15D greatly reduces the adhesion of HepG2 plated onto vitronectin of 66%; this result is even better than that obtained from the monoclonal anti-αvβ5 antibody (57%), used as positive control. Incubation of cells with the scrambled peptide (negative control) does not decrease cell adhesion. Considering the already reported RGDechi15D binding specificity towards αvβ5 with respect to αvβ3 [36], this result confirms the specificity of action of RGDechi15D towards αvβ5 on the HepG2 cell surface and indicates the ability of the peptide to compete with vitronectin and to prevent HepG2 cell adhesion. Furthermore, it is worth noting that the inhibition occurred in a concentration-dependent manner with an IC_50_ of 31.6 µM (Figure 2B). With the aim to evaluate the capability of the peptide to discriminate between αvβ5 and α5β1, cell adhesion assays on K562 cells, displaying α5β1 at high levels and αvβ3 and αvβ5 at very low levels [32], were carried out. The cells were seeded both onto fibronectin, the main ECM protein that binds α5β1 receptor, and onto α5β1 antibody coated plates. The results obtained indicate that RGDechi15D is not able to inhibit K562 adhesion neither on fibronectin nor that of the α5β1 specific antibody; in this experiment the RGDechi peptide [33] was used as a control to recognize αvβ3 integrin [32] thus proving the validity of experimental data (Figure 3A,B). The competition experiments here reported are all in the direction of corroborating RGDechi15D specificity towards αvβ5 with respect to αvβ3 or α5β1 integrins.

### 2.3. Effect of RGDechi15D on HepG2 Migration and Invasion

**Figure 4:**
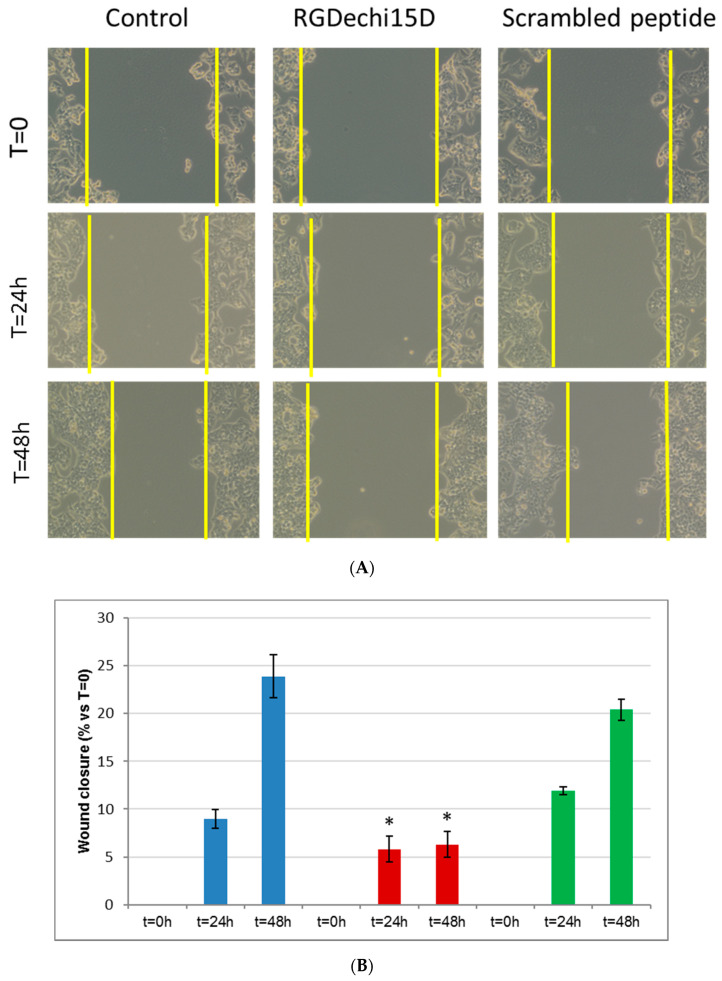
Effect of RGDechi15D on wound healing activity. (**A**) The wound closure was determined by the scratch assay. After the treatment, the cells were photographed using phase-contrast microscopy at 0, 24, and 48 h. (**B**) Quantification of wound closure. Graphic represents the wound width for each treatment, expressed as the mean ± SE of the percentage of the wound width at 24 or 48 h with respect to t = 0. The means resulted from at least three independent experiments performed in triplicate. Statistical significance was analyzed using Student’s t test, unpaired, two-sided (* *p* < 0.05). Blue: control; Red: RGDechi15D; Green: scrambled peptide.

**Figure 5:**
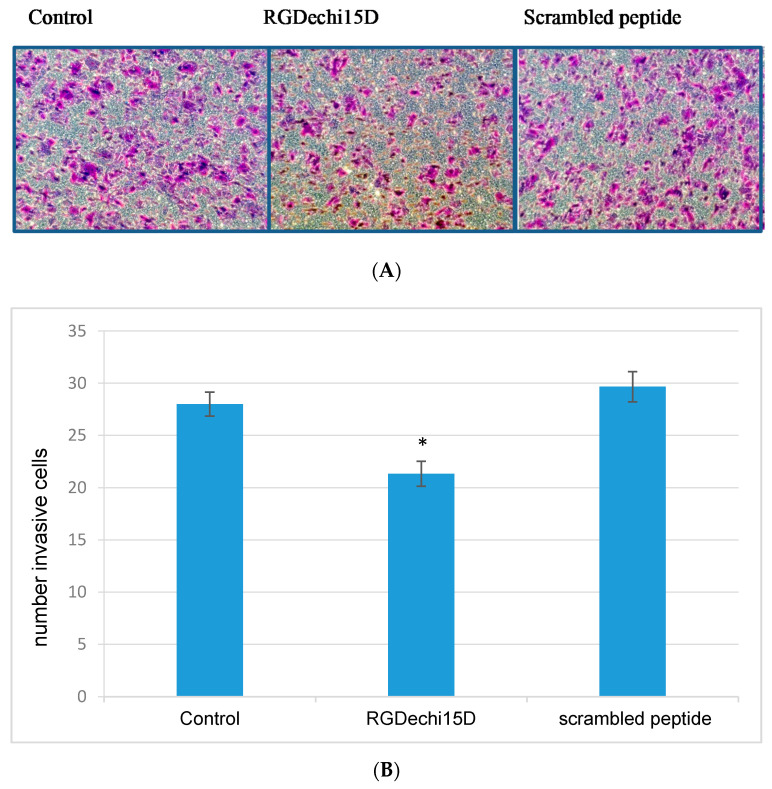
Inhibition of HepG2 invasion by RGDechi15D. (**A**) The invasion of HepG2 cells was assessed in trans-well chambers coated with ECL Cell Attachment. Invaded cells were fixed, stained, and captured at 20× magnification. The picture is representative of three independent experiments performed in duplicate. (**B**) The invasive HepG2 cells were counted and their number (± SE) was reported in the scheme (* *p* < 0.05).

The major life-threatening event in cancer patients is the metastasis formation which involves different events, such as cell migration and invasion into blood or lymphatic vessels in distal organs. Firstly, to investigate whether RGDechi15D could interfere with these mechanisms, an in vitro scratch assay was performed on HepG2 cells. After 24 h of seeding, the cell monolayers were scratched linearly and incubated with the peptide RGDechi15D (50 μM). Then, images were taken at 0, 24, and 48 h after wounding (Figure 4A). The results show that in the presence of the peptide, the wound healing was delayed compared to scrambled peptides and untreated cells. Remarkably, RGDechi15D inhibits closure of the gap both at 24 and 48 h after the scratch (Figure 4B). Afterwards, to evaluate the cell invasion inhibition, HepG2 cells were incubated with 50 µM peptide for 18 h and seeded on trans-well chambers coated with ECL Cell Attachment, an efficient model system that better mimics in vivo cell invasion. As shown in Figure 5A, significant decrease in tumor cell invasiveness is observed when cells are treated with the RGDechi15D peptide with respect to untreated cells (control) or scrambled peptides. The decrease in invasiveness was quantified and resulted in 20%, as indicated in the graph (Figure 5B). The effect on cell adhesion, migration, and invasion, events closely correlated with the metastatic cascade, highlight promising features of this peptide in inhibiting critical steps of cancer progression.

### 2.4. Evaluation of RGDechi15D Capability to Inhibit New Vessels Formation

Angiogenesis is required for invasive tumor growth and metastasis and constitutes a key process in the control of cancer progression, thus elucidating its tight correlation with tumor cell invasion and migration. The involvement of αvβ5 integrin in all these events together with its high expression in HCC tissues are well documented. Since this tumor is highly aggressive and often culminates in extensive metastasis [21,22,23,41,42], the targeting of integrin αvβ5 is a promising approach to improve the survival rate.

**Figure 6:**
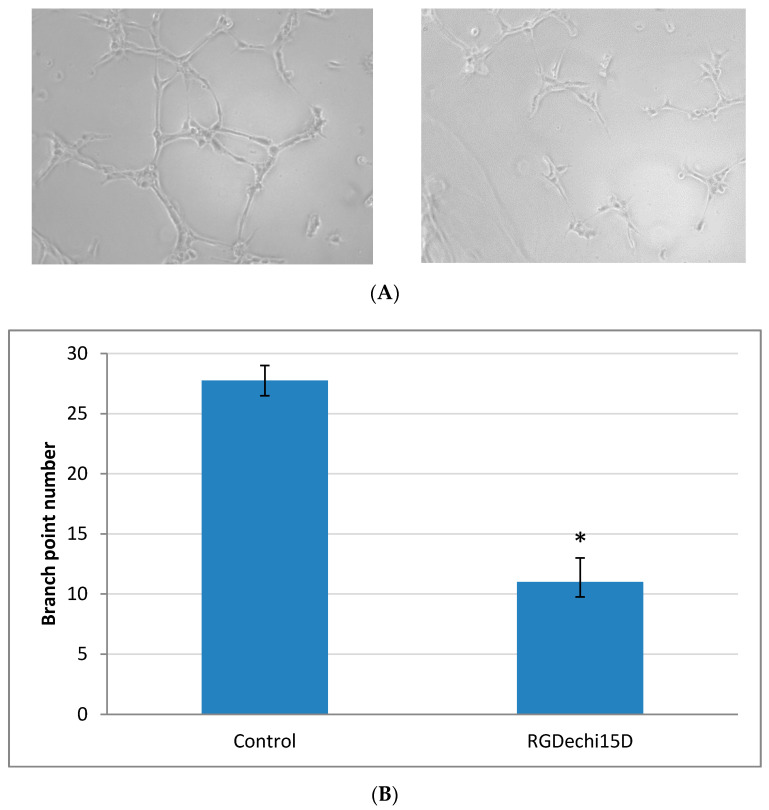
Angiogenesis Assay. The in vitro angiogenesis assay, using extracellular matrix (ECM) was performed to characterize the anti-angiogenic effect mediated by treatment with the RGDechi15D peptide. (**A**) Representative phase-contrast micrographs of tubular structures in cultured Human umbilical vein endothelial cells (HUVEC) previously exposed to 50 μM RGDechi15D peptide. Magnification: 10×. (**B**) The bar graph illustrates the significant decrease in the percentage of branch points after RGDechi15D treatment compared to control cells. Data are shown as mean (± SE) of two independent experiments performed in duplicate (* *p* < 0.05).

The ability to inhibit the generation of new capillary blood vessels by the RGDechi15D peptide was examined by an angiogenesis in vitro assay. Human umbilical vein endothelial cells (HUVEC) were used as specific system to evaluate tube assembly. The cells were incubated on a gel containing various matrix proteins such as laminin, collagen type IV, heparan sulfate. Cellular network structures were already developed by 6 h. In Figure 6A the formation of new capillary tubes is evident and, interestingly, in the sample treated with RGDechi15D results to be significantly reduced. The branch points were counted to better evaluate the percentage of inhibition. The results indicate that the RGDechi15D peptide is able to inhibit the branch point formation of about 60% (Figure 6B).

### 2.5. Effect of RGDechi15D on Cell Proliferation

**Figure 7:**
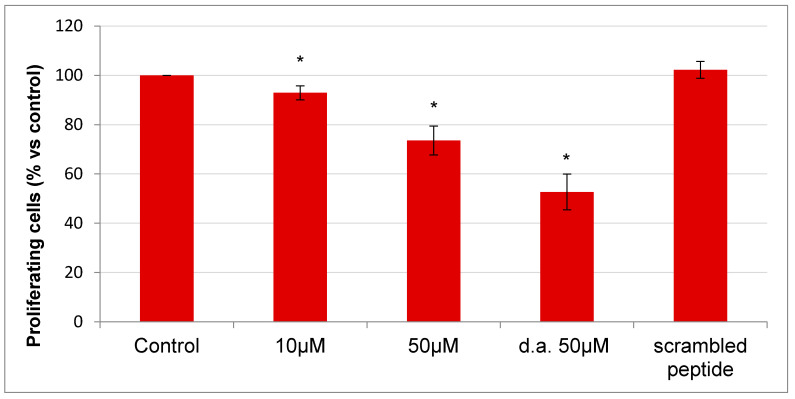
Proliferation assay. HepG2 were treated with the peptide at different concentrations. Cell proliferation was evaluated considering the normalized cell index (CI) of each curve at about 24 h after treatment; results are presented as the percentage of CI of adherent cells versus control (cells not treated). HepG2 were also treated with 50 µM RGDechi15D with a second addition of 50 µM peptide (d.a. = peptide double addition) after 6 h. The results are expressed as means ± SE of at least three independent experiments performed in triplicate. Statistical significance was analyzed using Student’s t test, unpaired, two-sided (* *p* < 0.05).

To assess if RGDechi15D has cytotoxic activity besides ability to interfere with the initial stages of tumor dissemination such as adhesion, invasion and angiogenesis, we evaluated the peptide effect on cell proliferation and on related pathways. To this aim, HepG2 were thus incubated with the peptide at different concentrations (10–50 µM) for 24 h; furthermore, 6 h after the first incubation, one cell aliquot was incubated with a second addition of peptide (50 µM). As indicated in Figure 7, RGDechi15D inhibits cell proliferation in a dose-dependent manner; in particular, at the higher concentration used, RGDechi15D induces inhibition of proliferation (27%) with respect to the untreated cells. The scrambled peptide has no effect on cell proliferation. An interesting result was obtained by the double addition of the peptide, which results in a greater decrease in cell proliferation (47%) suggesting a probably poor serum stability of RGDechi15D.

### 2.6. Quantitative Analysis of the AKT Phosphorylation

AKT is an important effector of cell survival whose phosphorylation determines the activation of PI3K pathway triggered by integrin signaling [43].

**Figure 8:**
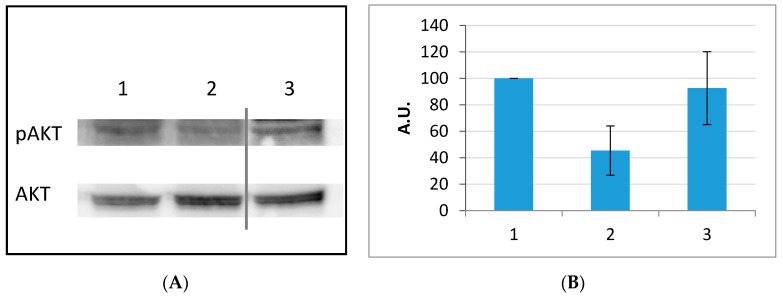
Western Blotting of pAKT. Analysis of the AKT phosphorylation state after peptide treatment was carried out on HepG2. Cells were treated with the RGDechi15D peptide for 1 h. (**A**) Total cellular extracts (50 μg) were resolved by SDS PAGE and analyzed by Western blotting with anti-pAKT (Ser473) and anti-AKT antibodies. The Western blotting is representative of three independent experiments. (**B**) Bar graph illustrates the percentage of band quantification respect to the control. 1 = Control, 2 = RGDechi15D, 3 = Scrambled peptide. The results are expressed as mean ± SE of percentage of pAKT normalized to the density of AKT.

The effect of RGDechi15D binding on the activation of the integrin receptor was analyzed in light of the results obtained with the anti-proliferative assay. For this purpose, the phosphorylation of AKT was evaluated by Western blotting analysis performed on HepG2 cell lysates after treatment with 50 µM RGDechi15D for 1 h. In Figure 8, a decreased signal level of pAKT with RGDechi15D treatment compared to not treated cell lysates can be observed. No effect on the signal is induced by scrambled peptide treatment. This result clearly suggests that RGDechi15D can reduce AKT phosphorylation in HepG2 cell line in good agreement with its capability to reduce the cell proliferation as well.

### 2.7. Determination of Caspase-3 Activity 

**Figure 9:**
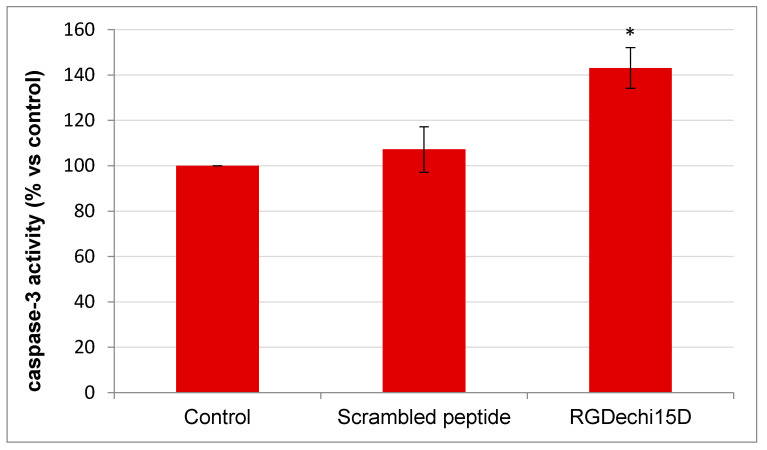
Apoptosis assay. Caspase-3 activity on HepG2 was determined after incubation with peptides (50 µM) for 6 h. Caspase-3 activity (calculated as nmol 7-Amino-4-trifluoromethylcoumarin (AFC)/min/µg protein) was expressed as the percentage of caspase-3 activity with respect to control (untreated cells). The results are expressed as means ± SE of at least three independent experiments performed in triplicate. Statistical significance was analyzed using Student’s t test, unpaired, two-sided (* *p* < 0.05).

Finally, to investigate if cell death induced by the RGDechi15D effect is due to the apoptosis process, a caspase-3 activity assay was carried out. HepG2 cells were treated with 50 µM peptide for 6 h and, as shown in the Figure 9, the examined peptide induces a remarkable increase in caspase activity, the differently scrambled peptide does not induce apoptosis, thus suggesting that RGDechi15D has a direct effect on cell viability mediated by an apoptotic pathway. These results are in good agreement with several reports demonstrating that cell detachment caused by the treatment with RGD-based peptides determines cell death by apoptosis, a process known as anoikis [44].

## 3. Conclusions

Here, we demonstrate that in HepG2 cell line the RGDechi15D peptide shows an interesting effect in some steps of the metastatic pathway. In addition, the peptide displays cytotoxic activity, ascribable to the interference with the PI3K pathway, mediated by an apoptotic process, in accordance with other RGD based peptides that, through cell detachment, send cells into apoptosis. Collectively, the obtained results give indications about a potential role of RGDechi15D as a selective ligand for HCC targeting.

Moreover, considering that αvβ5 is an interesting player in different pathological events, ranging from tumor to viral diseases, selective antagonists could be considered appealing candidates for the treatment of a wide spectrum of disorders.

## 4. Materials and Methods

### 4.1. Cell Lines and Culture Conditions

Human hepatocarcinoma cell line (HepG2) (ATCC U.S.) were grown in DMEM supplemented with 10% fetal bovin serum (FBS), 1% glutamine, 100 U/mL penicillin and 100 µg/mL streptomycin (Euroclone, Milan, Italy). Human umbilical vein endothelial cells (HUVEC) were purchased from Lonza. Cells were grown in endothelial cell growth medium (EGM-2) from Lonza, (Basel, Switzerland). All experiments were performed using low passage cell cultures [45]. Chronic myelogenous leukemia cells line (K562) (ATCC, Manassas, VA, USA.) were grown in RPMI with added heat-inactivated 10% FBS (fetal bovine serum), 2 mM glutamine, 100 U/mL penicillin and 100 µg/mL streptomycin (Euroclone, Milan, Italy). The cells were maintained in humidified air containing 5% CO_2_ at 37 °C.

### 4.2. FACS Analysis for αvβ5 and αvβ3 Integrins

For FACS analysis adherent cells at about 70% confluence were detached using 0.1 mM EDTA in PBS (Sigma Aldrich, Darmstads, Germany), centrifuged and re-suspended in PBS containing 0.2% BSA. Cell aliquots (2.5 × 10^5^ cells) were treated with primary monoclonal antibody PE conjugate, (Millipore, Burlington, MA, USA) or isotype control (Santa Cruz Biotechnology, Dallas, TX, USA), at the same concentration (14 µg/mL), whole a reaction volume of 50 µL for 30 min at 4 °C. After washing, the cells were analyzed by using a flow cytometer equipped with a 488 nm argon laser (FACScan, Becton Dickinson, Franklin Lakes, NJ, USA). A total of 20,000 events per sample were collected. Integrin quantification with Quantibrite PE beads (BectonDickinson Biosciences) was evaluated as previously described [46]. Values of fluorescence intensity were obtained from the histogram statistic of CellQuest software.

### 4.3. Cell Adhesion Assay

The effect of peptides on HepG2 or K562 adhesion was tested on NUNC MaxiSorp 96 well plates (Dasit Sciences, Milan, Italy) coated with 10 μg/mL vitronectin (for HepG2), fibronectin or anti-α5β1 antibody (for K562) (Millipore, Burlington, MA, USA). 15,000 cells/well were incubated in the presence of peptides (50 μM) or antibodies (10 μg/mL) at 4 °C for 30 min as previously reported [36] and then cells were seeded on coated plates for 1 h. Non adherent cells were gently removed by repeated washing and adherent cell number was evaluated by crystal violet (Sigma Aldrich) assay, which correlates optical density with cell number. For the IC_50_ calculation, the peptides were added at different concentration starting from 1 μM to 125 μM. The mean value ± standard error (SE) of adherent cells for each treatment was expressed as relative percentage of cell number vs. untreated cells (control). Statistical differences were determined by Student’s t test, paired, two-sided. All experiments were performed in triplicate and repeated at least 3 times; a *p* value < 0.05 was considered to be significant. The IC_50_ value was calculated by GraphPad Prism software.

### 4.4. In Vitro Scratch Assay

HepG2 cells were cultured in 6 well plates until they reached the confluence, and were linearly scratched with a plastic pipette tip to create a wound [47]. After washing with PBS, necessary to remove loose cells, the medium containing 50 μM of peptides was then added, and cells were incubated at 37 °C in a humidified incubator in 5% CO_2_. Each scratch area was photographed at 0, 24, and 48 h. The wound size for the different peptides was calculated as width at each end-points with respect to their value at 0 h as follows: wound closure (%) = 1 − (wound width t = x/wound width t = 0) × 100.

### 4.5. Invasion Assay

Cell invasion was assayed using transwell chambers coated with ECL Cell Attachment matrix (Millipore Corporation) used according to manufacturer’s instructions. Two duplicates were set for the assay. Cells at the logarithmic phase were detached using 1 mM EDTA in PBS and washed twice with serum-free DMEM. A total of 1 × 10^5^ cells/150 μL were seeded in the upper chamber. In the lower chamber, 600 μL of DMEM medium containing 10% fetal bovine serum was added for incubation at 37 °C in a 5% CO_2_ incubator for 18 h. After removal of the upper chamber, they were washed twice with PBS and migrated cells were fixed with 10% formalin for 10 min. Next, the cells on the bottom surface of the membrane were stained with crystal violet for 30 min. The cells which did not migrate were removed with cotton swabs [47]. Cell images were obtained under a phase contrast microscope (Zeiss, Oberkochen, Germany) and the cells were counted by Axiovert 200 software (Zeiss). Transwell migration assays were repeated three times and performed in duplicate. All measurement data are presented as the mean ± SE. Statistical analysis was performed using a t-test where *p* < 0.05 was considered to indicate a statistically significant difference.

### 4.6. Angiogenesis Assay

ECMatrix^TM^ solution (100 µL) was plated in a pre-cooled 96 well plate and incubated for 1 h at 37 °C. HUVEC cells were harvested, incubated (10000 cells) with 50 μM peptide and seeded onto solidified matrix. After 6 h of incubation the tube formation was inspected under an inverted light microscope at 10× magnification [48]. Imagines were acquired by Axiovert 200 Zeiss microscopy fluorescence. The experiments were performed two times in duplicate.

### 4.7. Proliferation Assay

The effect of peptides on HepG2 proliferation was analyzed with an RTCA iCELLigence™ instrument (ACEA, San Diego, CA, USA) with a method that uses label-free, real time, non-invasive analysis [49]. The instrument records a signal of electrical impedance due to the cell covering of gold electrodes located on the surface of a special microplate. The variation of the electrical impedance is named cell index (CI). CI values are proportional to the area of cells adherent and, as a consequence, to their numbers [50]. In detail, 40,000 cells/well were seeded and incubated in the presence of peptides for 24 h. The CI was calculated by measuring the slope of the proliferation curve between two selected time points for each curve, considering that the maximum CI values observed is the end-point of the experiments. Statistical differences were determined by Student’s t test, paired, two-sided. All experiments were performed in triplicate and repeated at least three times; a *p* value less than 0.05 was considered to be significant.

### 4.8. AKT Phosphorylation by Western Blotting

HepG2 cells were incubated with peptides (50 μM) for 1 h at 37 °C. Whole cell lysates were obtained by using lysis buffer (50 mM Hepes, pH 7.4, 50 mM NaCl, 1% Triton) supplemented with phosphatase (Sigma Aldrich, Milan, Italy) and protease inhibitor cocktails (Roche, Milan, Italy).

Cell lysates were incubated on ice for 30 min and then centrifuged at 13.000 rpm for 30 min to remove cell debris. Protein concentrations were determined by the Bradford method using Bio-Rad reagent (Bio-Rad Laboratories, Hercules, CA, USA). Proteins (50 μg) were resolved by SDS-polyacrylamide gel electrophoresis (SDS-PAGE) and transferred to a PVDF membrane (Millipore). The membrane was probed with the primary antibodies (anti pAKT (Ser473) or anti AKT (Cell Signaling Technologies, Boston, MA, USA)) over night at 4 °C. Proteins were visualized with an enhanced chemiluminescence detection system (Euroclone, Milan, Italy) and images were acquired with ChemiDoc XRS System (Bio-Rad Laboratories, Italy) and analyzed with the QuantityONE software. Reblot was performed using a harsh stripping buffer containing 2% SDS, 60 mM Tris HCl, pH 6.8, 0.8% β-mercaptoethanol, incubating the membrane at 50 °C for 30 min with some agitation.

### 4.9. Analysis of Caspase-3 Activity

Determination of caspase-3 activity was performed by a fluorometric assay based on the proteolytic cleavage of the carbobenzoxy-Asp-Glu-Val-Asp-7-amino-4-methylcoumarin (Acetyl-DEVD-AFC Alexis Biochemicals, San Diego, CA, USA) as described elsewhere [45]. In detail, HepG2 were treated with the peptides (50 µM) for 6 h at 37 °C. After 6h, cells were processed with reaction buffer (50 mM HEPES, pH 7.5, 0.1 mM EDTA, 0.1% Nonidet P-40, 0.1% CHAPS and 1 mM DTT) and 20 µg of lysates were incubated with 20 µM Ac-DEVD-AFC at 37 °C for 1 h. Samples were analyzed using a microplate reader (BioTek, Winooski, VT, USA) (excitation wavelength 360 nm; emission wavelength 528 nm). An AFC standard curve was determined, and caspase-specific activity was calculated as nmol of AFC produced per min per µg proteins at 37 °C at saturating substrate concentration (20 µM).
